# Obesity and Colorectal Cancer: A Narrative Review

**DOI:** 10.3390/medicina60081218

**Published:** 2024-07-27

**Authors:** Bárbara Cristina Jardim Miranda, Francisco Tustumi, Eric Toshiyuki Nakamura, Victor Haruo Shimanoe, Daniel Kikawa, Jaques Waisberg

**Affiliations:** 1 Department of Surgery, Instituto de Assistência Médica ao Servidor Público Estadual—IAMSPE, Sao Paulo 04029-000, SP, Brazil; 2 Department of Surgery, Faculdade de Medicina do ABC—FMABC, Santo Andre 09060-870, SP, Brazil; 3 Department of Gastroenterology, Faculty of Medicine, Universidade de São Paulo—USP, Sao Paulo 14040-903, SP, Brazil; 4 Department of Health Sciences, Sociedade Beneficente Israelita Brasileira Albert Einstein, Sao Paulo 05652-900, SP, Brazil

**Keywords:** obesity, bariatric surgery, colorectal neoplasms

## Abstract

*Background and Objectives*: Cancer is a multicausal disease, and environmental, cultural, socioeconomic, lifestyle, and genetic factors can influence the risk of developing cancer. Colorectal cancer (CRC) stands as the third most common cancer globally. Some countries have observed a rise in the incidence of CRC, especially among young people. This increase is associated with lifestyle changes over the last few decades, including changes in diet patterns, a sedentary lifestyle, and obesity. Currently, obesity and overweight account for approximately 39% of the world’s population and increase the risk of overall mortality of certain cancer types. This study aims to conduct a literature review examining the association between obesity and CRC. *Materials and Methods*: This narrative review explored the pathophysiological mechanisms, treatment strategies, and challenges related to obesity and CRC. *Results*: Several studies have established a clear causal relationship between obesity and CRC, showing that individuals with morbid obesity are at a higher risk of developing colorectal cancer. The adipose tissue, particularly the visceral, secretes proinflammatory cytokines, such as TNF-alpha, interleukin-6, and C-reactive protein. Chronic inflammation is closely linked to cancer initiation and progression, with a complex interplay of molecular mechanisms underlying this association. Obesity can complicate the treatment of CRC due to several factors, reducing the therapeutic effectiveness and increasing the risk for adverse events during treatment. Dietary modification, calorie restriction, and other types of weight-control strategies can reduce the risk of CRC development and improve treatment outcomes. *Conclusions*: Obesity is intricately linked to CRC development and progression, making it a crucial target for intervention, whether through diet therapy, physical exercises, medical therapy, or bariatric surgery.

## 1. Introduction

### 1.1. Background

Cancer is a multicausal disease, and environmental, cultural, socioeconomic, lifestyle (obesity, smoking, alcohol consumption, physical inactivity, and unhealthy diet), and genetic factors are associated with cancer development [1]. Globally, demographic and epidemiological studies indicate the increasing significance of cancer in the forthcoming decades. Currently, cancer ranks as the second cause of mortality. It is projected that by 2025, the burden of cancer will surge by 50% [1]. The top ten cancer types represent more than 60% of total new cases. Female breast cancer is the most common in the world, with 2.3 million (11.7%) new cases, followed by lung cancer, with 2.2 million (11.4%), colon and rectum, with 1.9 million (10%), prostate, with 1.4 million (7.3%), and non-melanoma skin cancer, with 1.2 million (6.2%) new cases [2].

The incidence of cancer is rising rapidly worldwide [3]. This escalation is primarily attributed to demographic shifts. There has been a decline in fertility and infant mortality rates in the last decades, leading to a consequent increase in the proportion of elderly individuals. Concurrently, from an epidemiological transition perspective, there is a gradual shift from mortality caused by infectious diseases to deaths related to chronic diseases. Aging populations, coupled with changes in behavior and the environment, including structural alterations impacting mobility, recreation, diet, and exposure to environmental pollutants, are conducive to an increase in both cancer incidence and mortality [4].

Colorectal cancer (CRC) stands as the third most common globally, affecting both men and women [5]. The estimated number of new cases of colon and rectal cancer in Brazil for each year is 45,630 cases, corresponding to an estimated risk of 21.1 cases per 100,000 inhabitants, with similar rates for men and women [2].

Some countries have observed a rise in the incidence of CRC among young people. This increase is linked to lifestyle changes over the last few decades, including a sedentary lifestyle, obesity, and low intake of fiber, fruits, vegetables, and lean meats [2]. Although observational studies consistently show these associations, the causal relationship has only started to be investigated in recent years.

A combination of environmental and genetic factors interplays for CRC development. While inherited susceptibility significantly increases the risk, most CRC cases are sporadic rather than familial. Key factors influencing susceptibility include hereditary CRC syndromes (such as Lynch syndrome and adenomatous polyposis syndromes), a personal history of CRC or adenomatous polyps of the colon, inflammatory bowel disease, and age, particularly after 45 years old [5].

One of the CRC risk factors has stood out in the last decades. In 1940, Albert Tannenbaum established a significant association between body weight and cancer incidence in a rodent animal model. Tannenbaum showed that mouse skin tumors, induced either by ultraviolet light or carcinogenic hydrocarbons, formed in more significant numbers and earlier in mice receiving a high-fat diet than in control mice consuming the basal rations [6]. This association was not formally studied in epidemiological studies until 1959, when the American Cancer Society conducted a long-term prospective analysis on 750,000 subjects. The analysis revealed that mortality attributable to cancer was higher for individuals who were 40% above the average weight [7].

Obesity is an increasing global health problem. Since 1980, the prevalence of obesity has nearly doubled, and currently, more than half a billion of the world’s adult population are obese [8]. Obesity is a phenomenon observed in practically all age groups of the population in numerous countries worldwide. Once more prevalent among adults, it now also significantly affects children and adolescents. Currently, obesity and overweight account for approximately 39% of the world’s population and increase the risk of overall mortality of certain cancer types [9]. 

### 1.2. Rationale and Knowledge Gap

Knowledge about obesity and its relationship with CRC, including management, prevention measures, and treatment, still harbors uncertainties. Understanding the intricate interplay of these elements is vital for developing comprehensive prevention and therapeutic recommendations.

### 1.3. Objective

Our study aims to conduct a literature review examining the association between obesity and colorectal cancer. This review explores the pathophysiological and molecular mechanisms, treatment options, and challenges related to obesity and CRC.

## 2. Methods

This narrative review examined the association between obesity and CRC. The review explored the pathophysiological and molecular mechanisms, treatment strategies, and challenges related to obesity and CRC. The search encompassed databases such as PubMed, Embase, Lilacs/BVS, Cochrane Central, and Google Scholar, covering the inception of these databases until February 2024. Only English and Portuguese studies were considered for inclusion. Search terms comprised “cancer”, “neoplasm”, “tumor”, “oncogenesis”, “oncology”, “obesity”, “obese”, “overweight”, “insulin resistance”, “metabolic syndrome”, “colorectal”, “colonic”, “colon”, “rectal”, and “rectum”. The review encompassed observational and experimental studies, including human studies, in vivo and in vitro studies, and animal models (see Table 1).

## 3. Results

### 3.1. Included Publications

After screening and selecting relevant studies, 83 articles [1,2,3,4,5,6,7,8,9,10,11,12,13,14,15,16,17,18,19,20,21,22,23,24,25,26,27,28,29,30,31,32,33,34,35,36,37,38,39,40,41,42,43,44,45,46,47,48,49,50,51,52,53,54,55,56,57,58,59,60,61,62,63,64,65,66,67,68,69,70,71,72,73,74,75,76,77,78,79,80,81,82,83] were used for this review.

### 3.2. Obesity and Incidence of Colorectal Cancer

Obesity stands as a significant risk factor for various solid cancers, including hormone-related neoplasms, esophageal cancer, and CRC [10]. Past reviews have shown that obesity imposes a 60% higher risk of CRC compared to individuals of normal weight [9]. Several studies have established a clear causal relationship between obesity and CRC, showing that individuals with severe obesity (body mass index (BMI) ≥ 30 kg/m^2^) are at a higher risk of developing CRC (risk ratio (RR) = 1.93; 95% confidence interval (CI): 1.15–3.25). Maternal obesity also increases the risk of CRC development for the offspring (adjusted hazard ratio (aHR) = 2.51; 95% CI: 1.05–6.02) [11].

A cross-sectional study conducted in Romania found a significant association between obesity and CRC. Obese patients had a higher prevalence of rectal cancer compared to normal-weight patients (BMI 18.5–24.9 kg/m^2^), with an odds ratio of 1.56 (*p* = 0.0213) [12]. A study by Pang et al. revealed that central adiposity, as measured by waist circumference and waist-to-hip ratio, is positively associated with CRC risk [13]. Other measures, including hip circumference, percent body fat, height-adjusted weight, weight-to-height ratio, and weight change, were also positively associated with CRC risk [14]. The excess body weight contributes to approximately 5% of incident colorectal cancer cases only in the United States of America [15].

In a prospective international population-based study of 580,000 people assessing metabolic syndrome and cancer risk, after 12 years of follow-up, 2834 men and 1861 women had been diagnosed with CRC (RR = 1.25 for men, 1.14 for women) [16]. Sex differences in the age of onset of metabolic syndrome or the effects of sexual hormones on cell proliferation might explain this gender difference.

Due to the significant association between obesity and CRC, it is reasonable to recommend that obese and overweight patients should undergo a special screening program for CRC detection. These patients may benefit from starting screening earlier and having shorter intervals between screening exams.

### 3.3. Obesity and Colorectal Cancer Prognosis

Several studies suggest that, in addition to a higher cancer incidence, patients with obesity have CRC with a poorer prognosis. A cross-sectional study examined the relationship between CRC oncologic stage and BMI, revealing that BMI is a determinant in cancer progression. Patients with higher BMIs had a higher incidence of CRC in stage III (*p* = 0.02) [12].

However, while obesity and high BMI are associated with poorer survival rates among CRC patients, maintaining a moderately high BMI can be beneficial for nutritional reserve and survival, particularly for patients with advanced CRC at risk of malnutrition. Aparicio et al. found that patients with CRC in the BMI range of 28–30 kg/m^2^ had a better prognosis than normal-weight individuals, possibly due to greater lean muscle mass in overweight patients, which may improve their condition [17]. Conversely, a low BMI is linked to an increased risk of death, a poorer prognosis, and malnutrition in CRC patients. Those with a very low BMI or consistently declining BMI may be more prone to complications or death due to nutritional deficiencies or muscle loss. Cachexia, sarcopenia, and loss of adiposity in patients with stage III or IV tumors can contribute to a poor prognosis, indicating that underweight and weight loss may lead to tumor progression and worse outcomes. Interaction between underweight and cachexia, both poor prognostic factors in CRC patients, underscores the importance of weight management after diagnosis for treatment outcomes [18]. 

At a systemic level, cancer can alter the host metabolism by inducing profound changes in nutrient intake, culminating in cachexia [19]. Cancer cachexia (CC) is a wasting syndrome that occurs in up to 80% of cancer patients. Tumor characteristics, such as tumor proliferation rates and inflammatory status, contribute to CC. Interleukin (IL)-6, the activator of transcription (STAT) 3 signaling, and mitogen-activated protein kinase (MAPK/ERK) are linked to CC, leading to skeletal muscle wasting, cardiac dysfunction, and hypothalamic inflammation, impairing prognosis in patients with CC [19,20]. Patients with CC have increased lipolysis, increasing free fatty acids. Cytosolic free fatty acids are transferred to the mitochondrial matrix, where they are used for β-oxidation, thereby waning fat storage. CC is closely implicated with liver metabolic and cardiac dysfunctions, as cancer-induced proinflammatory cytokines play a crucial role in regulating metabolic homeostasis, such as energy production and glycogen storage [19,20]. 

This U-shaped relationship between BMI and patient outcomes suggests that the management of BMI in CRC patients should be careful, and a specialized nutritional team should guide any strategy for weight loss in patients with obesity. 

### 3.4. Mechanisms for the Association between Colorectal Cancer and Obesity

**Figure 1:**
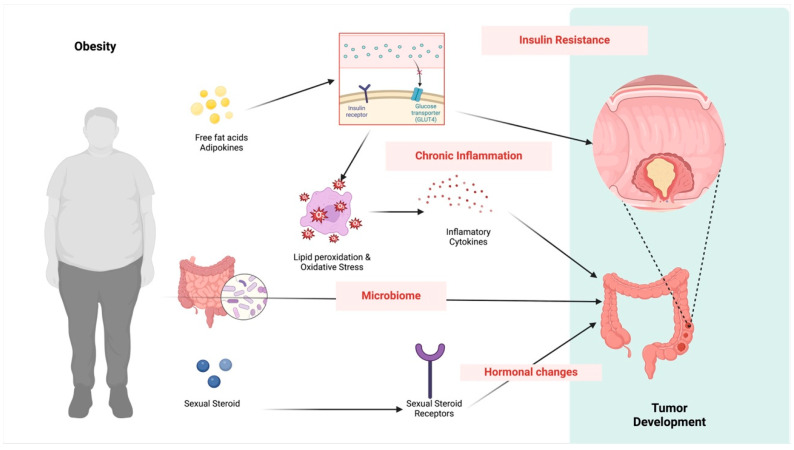
Association between inflammation from obesity and cancer.

Several mechanisms can explain why obesity is associated with a higher incidence of CRC and poorer prognosis, primarily involving metabolic changes and chronic inflammation (see Figure 1). 

There is an established association between inflammation and cancer. The inflammation of the metabolic syndrome is a subclinical systemic inflammation initiated by adipose tissue [7]. Adipose tissue, traditionally viewed solely as a passive energy storage site, is now recognized as an active endocrine organ that releases a variety of adipokines, cytokines, and other signaling molecules [21]. In patients with obesity, the expansion of adipose tissue induces alterations in adipocyte function and surrounding tissues’ microenvironment, thereby fostering chronic low-grade inflammation.

Within adipose tissue, adipokines can locally stimulate inflammation and provoke insulin resistance. Additionally, adipocytes are pivotal in recruiting immune cells to adipose tissue. In obesity, there is a heightened infiltration of macrophages into adipose tissue, which further exacerbates the inflammatory response. These macrophages can be activated by factors derived from adipocytes and produce more proinflammatory cytokines, thus perpetuating a cycle of inflammation and immune cell recruitment [22]. Moreover, adipocytes may undergo cell death in response to stressors, such as hypoxia and nutrient overload, releasing damage-associated molecular patterns (DAMPs). These DAMPs can activate immune cells further, contributing to inflammation [23].

The adipose tissue, particularly the visceral, secretes proinflammatory cytokines, such as tumor necrosis factor-alpha (TNF-alpha), interleukin-6 (IL-6), and C-reactive protein (CRP). These cytokines are crucial in the pathogenesis of obesity-related complications, including insulin resistance, cardiovascular disease, and cancer. These inflammatory cytokines disrupt normal tissue homeostasis, promote insulin resistance, and increase oxidative stress, all contributing to the development and progression of obesity-related diseases. Additionally, adipose tissue dysfunction in obesity leads to the dysregulation of adipokine secretion, further exacerbating the inflammatory state and associated metabolic complications [7].

In individuals with obesity, the COX-2 gene is highly active in subcutaneous adipose tissue [24]. Arachidonic acid and PGE2 stimulate leptin release in obese adipose tissue [25]. Mice with a partially inactive COX-2 gene tend to become obese, suggesting a role for COX-2 in fat metabolism [26]. Obesity is associated with higher levels of cytokines, such as IL-6 and TNFα, which can boost COX-2 expression and PGE2 production [24]. Loss of prostaglandin (PG) production in adipose tissue increases lipolysis and energy expenditure, leading to resistance to diet-induced obesity. Studies suggest that COX-2 activity is linked to adipocyte differentiation, with COX-2 inhibition reversing the reduced expression of adipogenic markers in obese rats [27]. COX-2 synthesizes lipid mediators that promote inflammation and stimulate cell proliferation, angiogenesis, and immune suppression, all critical for cancer initiation and progression. 

Proinflammatory cytokines act on tissues and cells at the microenvironment level, which results in cancer development through direct and indirect effects on innate and adaptive immune cells, disordered tissue homeostasis, and increased oxidative stress. Chronic inflammation promotes DNA damage and chromosomal instability, which can lead to mutations, including mutations in the tumor suppressor gene p53, contributing to carcinogenesis. Chronic inflammation can upregulate the expression of vascular endothelial growth factor (VEGF) and hypoxia-inducible factor 1 (HIF-1), both of which are central to angiogenesis [28]. Chronic inflammation can activate the phosphoinositide 3-kinase (PI3K)/Akt pathway, which is involved in cell survival, proliferation, and metabolism. Activation of this pathway can confer a survival advantage to cancer cells, enabling them to evade apoptosis and promote tumor growth. Furthermore, chronic inflammation can dysregulate the mitogen-activated protein kinase (MAPK) pathway, leading to increased cell proliferation and survival. This pathway is crucial for transducing extracellular signals to the nucleus, thereby regulating gene expression involved in cell growth and differentiation [29].

Obesity-related inflammation in white adipose tissue increases proinflammatory mediators, contributing to insulin resistance (IR). Adipose tissue secretes various adipokines and cytokines, influencing endothelial and metabolic functions, leading to IR and the development of metabolic syndrome. Tissue sensitivity to insulin decreases by 30–40% when body weight exceeds 35–40% of the ideal weight. The association between IR and obesity may stem from chronic inflammation triggered by heightened synthesis and release of proinflammatory factors, such as TNF-α, IL-6, and C-reactive protein. These factors, acting via the insulin signaling pathway, contribute to IR in various organs and tissues. Furthermore, adipose inflammation alters adipokine concentrations, impacting IR and increasing insulin synthesis and release from pancreatic β-cells, resulting in hyperinsulinemia. 

Leptin, the first described adipokine, exerts pleiotropic actions [30]. After being actively transported across the blood–brain barrier, leptin induces a satiety effect within the arcuate nucleus of the hypothalamus, also known as “the satiety center”. This effect is mediated by an increase in anorexigenic peptides, such as proopiomelanocortin (POMC)/cocaine and amphetamine-regulated transcript (CART), alongside a decrease in orexigenic peptide synthesis, including neuropeptide Y (NPY)/agouti-related peptide (AgRP) [31]. In addition to regulating feeding behavior, leptin influences glucose and fat metabolism and regulates the endocrine system [32]. Numerous studies have highlighted leptin’s role in promoting cancer cell invasion, primarily through upregulating matrix metalloproteinase (MMP) expression [33]. Leptin facilitates invasion in cancer by increasing the expression of membrane type 1-matrix metalloproteinase (MT1-MMP), which correlates with disease stage and lymph node metastasis [34]. MMP-7, known for activating various MMPs, is associated with tumor progression in colorectal and ovarian cancers. Leptin enhances MMP-7 expression, promoting cancer cell migration and invasion via specific signaling pathways, while ObRb gene silencing suppresses leptin-induced MMP-7 expression [35]. In CRC, leptin-mediated MMP-7 expression and cell invasion involve MAPK/ERK and PI3K/AKT signaling pathways, further activating MMP-2 and MMP-9 and influencing cancer progression [36,37].

IR has been increasingly recognized as a risk factor for cancer development and progression. Among cancer patients, there is a marked prevalence of IR [38]. Epidemiological studies have demonstrated higher risks for several cancers in individuals with IR, including breast, colorectal, liver, pancreatic, endometrial, lung, hepatocellular, and prostate cancers, suggesting a close relationship between IR and carcinogenesis [39].

The mechanisms linking IR and cancer progression are complex and not fully understood. However, they involve aberrant insulin levels, insulin-mediated signaling, and the dysregulation of pathways, such as PI3K-AKT-mTOR and MAPK-RAS, crucial in controlling cellular functions, such as proliferation, gene transcription, and survival [40]. Insulin’s effects on the liver include increased synthesis of insulin-like growth factor 1 (IGF-1), which affects circulating IGF-binding protein levels. Changes induced by hyperinsulinemia may alter local bioavailable IGF-1 concentrations. 

Visceral obesity results in increased release of free fatty acids (FFAs), tumor necrosis factor a (TNFa), and resistin, and reduced release of adiponectin into the circulation, which leads to the development of IR and chronic hyperinsulinemia. Prolonged hyperinsulinemia reduces the production of IGFBP-1 and IGFBP-2 (which normally bind to and inhibit the action of IGF-I), with a resultant increase in the levels of free, ‘bio-active’ IGF-I, and concomitant changes in the cellular environment that favor tumor development [42]. Circulating IGF-I has been linked to an elevated risk of colorectal advanced adenomas and cancer [41]. This association is primarily attributed to the increase in free IGF-I levels, which, in turn, alters the cellular microenvironment, promoting mitogenesis and inhibiting apoptosis, thereby favoring tumor formation [42]. Several cellular actions of IGF-I favor tumor growth, including near-ubiquitous mitogenic action in human cells, inhibition of apoptosis, induction of hypoxia-inducible factor-1-mediated production of vascular endothelial growth factor (VEGF), induction of tumor-related lymphangiogenesis, and increased cell migration mediated by integrins and E-cadherin. IGF-I is important in the regulation of differentiation, cell size, and organization of the cellular cytoskeleton. Furthermore, IFG-I stimulates pathways that are key to early tumor initiation and potentiates the effects of other stimulators of cell growth, including estrogens [42].

Moreover, IR is associated with dysregulation of other metabolic pathways, such as increased lipolysis and free fatty acid release, which can further contribute to cancer development through mechanisms involving inflammation, oxidative stress, and altered cell metabolism [43].

Adipocytes play a central role in peripheral sexual hormone production, and changes in these hormones might also influence CRC carcinogenesis [44]. In vitro studies suggest that estradiol could promote colorectal cell tumorigenesis, primarily through its mitogenic effects mediated by intracellular receptors ERα and ERβ [45]. Similarly, studies in male mice indicate a potential role of testosterone in inducing colonic adenomas, possibly through its tumor-promoting effects. Another proposed mechanism involves increased stress hormones, such as cortisol, which could impact the tumor environment [46].

Finally, emerging evidence suggests a potential role of epigenetic alterations in linking obesity, the gut microbiota, and CRC. The gut microbiota is increasingly recognized as a crucial element influencing host metabolism and inflammation [47]. Obesity imposes changes in the gut microbiome, influencing appetite, inflammation, energy absorption, fat storage, and the circadian cycle [48]. Emerging research indicates that certain microorganisms and their byproducts may play a role in either promoting or inhibiting tumor formation through various mechanisms. Dysregulation of non-coding RNA (ncRNA) expression, mediated by the gut microbiome, has been implicated in the development of gastrointestinal cancers [49].

### 3.5. Challenges of Cancer Therapy in Patients with Obesity 

Obesity can complicate the treatment of CRC due to several factors, reducing the therapeutic effectiveness and increasing the risk for adverse events during treatment.

In radiotherapy, obesity imposes challenges with treatment planning and radiation delivery. The increased body size and altered body composition can make it difficult to accurately target tumors while sparing surrounding healthy tissue, potentially leading to increased side effects. Additionally, adipose tissue can create a physical barrier that reduces the effectiveness of radiation reaching the tumor [50].

In chemotherapy, obesity is associated with altered drug metabolism and distribution. Adipose tissue can act as a reservoir for chemotherapy drugs, reducing their circulating levels and potentially decreasing their efficacy. Moreover, individuals with obesity often require higher doses of chemotherapy, which can increase the risk of toxicity and side effects [51].

Chen et al. found that high levels of insulin conferred resistance to oxaliplatin in colon cancer cell lines, an effect that was reversed with concurrent PI3K inhibition, suggesting a reliance on aberrant Akt signaling. Chemo-resistant cells demonstrated an increased proliferative response to insulin with an accompanying decrease in insulin’s metabolic effects [52].

Considering the association between insulin and chemo-resistant cells, some medications, such as biguanides, theoretically could impact cancer’s response to chemotherapy. Metformin has been shown in some cancer cells to activate adenosine monophosphate-activated protein kinase, resulting in inhibition of the mTOR pathway, reducing tumor cell growth and proliferation, and possibly suppressing processes associated with chemotherapeutic resistance [52]. 

In addition, obesity is frequently related to poor preoperative fitness status, which has consistently shown an association with postoperative outcomes in abdominal surgery. Factors such as functional exercise capacity, mobility, perceived fatigue levels, and physical activity have been identified as independent predictors for a quicker recovery of physical functioning [53].

Visceral obesity poses significant challenges for surgery in CRC patients. Excessive visceral fat can obscure the surgical field, making visualization difficult during laparoscopic procedures and increasing the risk of intraoperative complications. Laparoscopic procedures are more challenging in patients with obesity compared to non-obese individuals. The thickening of the mesentery in obese patients can lead to challenges, such as vessel dissection and ligation difficulties, during laparoscopic surgery. The highly vascularized nature of adipose tissue in obese individuals also heightens the risk of bleeding during surgery [54]. Additionally, obesity can make it challenging to identify and dissect structures accurately, increasing the risk of inadvertent injury to surrounding organs. 

Surgical morbidity is higher in patients with obesity, including bleeding, infection, wound dehiscence, and anastomosis leakage. A systematic review indicated that obesity is linked to a longer hospital stay, with individuals with obesity requiring an additional day compared to non-obese counterparts. Additionally, operations on patients with obesity are approximately 20 min longer on average. The incidence of postoperative severe complications is higher among individuals with obesity, with rates around 21% compared to 15% in non-obese individuals. The risk of anastomotic leaks is notably increased in patients with obesity (RR = 3). Furthermore, patients with obesity are more than twice as likely to undergo conversion to open surgery compared to non-obese individuals [55,56]. Besides, patients with obesity often experience stoma-related issues. Individuals with a high BMI face increased risks of parastomal hernias and early skin irritation [54].

Finally, individuals with obesity may exhibit reduced responsiveness to palliative chemotherapy, particularly when targeted therapies are involved. A study of 120 patients with metastatic CRC undergoing bevacizumab-based therapy (*n* = 80) or chemotherapy alone (*n* = 40) found a significant correlation between high BMI and visceral and subcutaneous fat areas with a lack of response to bevacizumab-based treatment, but not in the chemotherapy-only group. Patients with higher BMI values had a notably shorter mean time to progression (TTP) than those with lower BMI values (9 vs. 12 months; *p* = 0.01) [27].

### 3.6. Obesity Treatment and Reduction in Colorectal Cancer Incidence

Obesity treatments are beneficial for preventing CRC development and improving outcomes of CRC treatment. Dietary modification, calorie restriction, and other types of weight-control strategies can reduce the risk of CRC development [54]. Weight loss has been shown to have a protective effect related to decreases in oxidative damage to DNA and RNA of white blood cells and an increasing telomere length of rectal mucosa samples. Additionally, the decreased systemic inflammation and hyperinsulinemia may contribute to its protective effect on the formation of CRC [57].

#### 3.6.1. Diet Therapy

Overweight and obesity stem from a disparity between energy intake and expenditure. A case-control study highlighted that individuals with CRC consume significantly more calories than the general population, contributing to their elevated BMI [54]. These patients often have a high-fat diet lacking fruits, vegetables, and dietary fiber. Such dietary patterns impact the profile of visceral adipose tissue and alter metabolic pathways. Therefore, individuals with a high BMI necessitate comprehensive dietary modifications for the prevention of CRC development and for treating patients with diagnosed CRC [58].

Western dietary habits notably elevate the susceptibility to distal colon and rectal tumors. The Western diet exerts a more pronounced impact on the progression of tumors characterized by KRAS wildtype and BRAF wildtype, displaying little to no CpG island methylator phenotype and exhibiting microsatellite stability [59].

An interventional study revealed that transitioning African Americans to a high-fiber, low-fat diet for two weeks led to a significant reduction in colonic mucosal inflammation and proliferation biomarkers associated with cancer risk. This effect was particularly evident through increased saccharolytic fermentation, butyrogenesis, and suppressed secondary bile acid synthesis. Secondary bile acids are closely linked to a pro-cancer effect, emphasizing the potential of dietary interventions in mitigating cancer risk factors [11].

Certain dietary patterns and nutrition can play a role in reducing the incidence of CRC. Foods rich in dietary fiber, garlic, milk, and calcium have shown evidence of reducing CRC incidence. Other foods with more limited evidence include non-starchy vegetables, fruits, foods containing folate, fish, selenium-containing foods, and foods containing vitamin D [60]. 

Fiber is a crucial substrate for gut bacterial fermentation, yielding beneficial metabolites known as short-chain fatty acids (SCFAs), including butyrate, acetate, and propionate. A growing body of evidence underscores the significant role of butyrate and other SCFAs in maintaining intestinal immune balance by modulating regulatory T cells (Tregs) [61]. Tregs play a central role in suppressing inflammatory and allergic responses by limiting the proliferation of effector CD4+ T cells. Butyrate and propionate have been shown to promote the extrathymic generation and functional differentiation of Tregs, offering protection against colitis [62]. These effects are mediated through various mechanisms, such as inhibiting histone deacetylase, enhancing an anti-inflammatory phenotype in colonic macrophages and dendritic cells via GPR109a activation, and inducing T-cell intrinsic epigenetic upregulation of the Foxp3 gene, a master regulator of Treg function [63]. Increased fiber intake has been shown to enrich specific gut bacteria, including *Lactobacillus* spp. and butyrate-producing strains, such as *Bifidobacterium, Clostridium, Anaerostipes, Eubacterium,* and *Roseburia* species. This heightened fiber consumption leads to an increased production of SCFAs in the gut [64].

Caloric restriction (CR), generally defined as a 10–40% reduction in caloric intake without a reduction in dietary nutritional content [68], can decrease clinical markers associated with cancer [65]. Initially viewed as limiting nutrients and metabolism, CR is now recognized for actively reducing oxidative stress and triggering various metabolic adjustments [66]. These include decreasing growth factors and anabolic hormones, boosting antioxidant defenses to counter DNA damage, lowering inflammation by reducing proinflammatory cytokines, and increasing corticosteroids and adiponectin levels [67]. CR also delays age-related declines in immune function and upregulates genes involved in DNA repair. Autophagy, a pivotal response to calorie restriction, is regulated by pathways sensing cellular energy and nutrient levels, such as AMP-activated kinase (AMPK), hexokinase 2 (HK2)–mTOR complex 1 (mTORC1), and protein kinase B (AKT)–mTORC1 pathways [68]. Autophagy is a vital self-degradative cleanup process that facilitates the removal of misfolded or aggregated proteins, as well as the recycling of damaged cell components. As a consequence, autophagy plays a substantial role in the prevention of a wide variety of diseases and, conversely, deregulated autophagy is known to be associated with several disorders, including metabolic diseases, neurodegenerative disorders, infectious diseases, and cancer [68]. The impaired autophagy activity leads to cancer cells’ resistance to cellular stress. In addition, tumorigenesis occurs due to disturbances in cell growth and genome instability as a consequence of autophagy inhibition [68]. 

As an alternative to CR, intermittent fasting has become a significant research focus in recent years for losing weight. During fasting, the levels of several anabolic hormones, such as insulin, IGF-1, and leptin, drop. Along with a reduction in metabolic fuel availability, these changes decrease anabolic signaling in noncancerous cells, leading to increased mTOR activity and decreased AKT activity. These signals slow cell growth and proliferation and can induce autophagy [19,68,69,70]. 

Nevertheless, there has yet to be a consensus regarding the best strategy to lose weight in cancer patients. A systematic review and meta-analysis compared the efficacy of caloric restriction, the ketogenic diet, and intermittent fasting on cancer initiation, progression, and metastasis. The study concluded that caloric restriction and the ketogenic diet were highly effective as anticancer therapies. However, a significant number of studies found no association between intermittent fasting and cancer modulation [69]. However, the response to diet is likely influenced by the patients’ individual characteristics, cancer types, and treatment regimen [70].

#### 3.6.2. Physical Exercises

Engaging in regular physical activity can have a profound impact on reducing the risk of CRC development. Physical activity helps maintain a healthy weight and reduce obesity. Additionally, exercise can improve insulin sensitivity and regulate hormone levels, such as insulin and estrogen, which can influence CRC risk. Physical activity also reduces inflammation and oxidative stress, which promote cancer development [72]. A randomized controlled clinical trial implemented a 12-month intervention of moderate-to-vigorous-intensity exercise, focusing on male participants who maintained an average of ≥250 min/week [73]. This study revealed significant decreases in colon crypt cell proliferation levels. 

#### 3.6.3. Medical Therapy

Anti-obesity drugs are critical in reducing IR and treating obesity, factors associated with CRC risk. These drugs act by reducing hepatic glucose production and stimulating glucose uptake by peripheral tissues, effectively lowering blood glucose levels in patients with obesity [74].

Calderon et al. examined the effectiveness and side effects of FDA-approved anti-obesity medications. The most prescribed medication was phentermine/topiramate extended-release, followed by liraglutide, bupropion/naltrexone sustained-release, and lorcaserin. The results showed that patients experienced a significant percentage of total body weight loss (%TBWL) at various time points, with 60.2% achieving at least 5% TBWL. Adverse events were reported in 22.4% of patients, leading to 9% discontinuing medication due to side effects [75].

Shaw et al. emphasized the significant role of AMP-activated protein kinase (AMPK) activation in metformin’s ability to lower mouse glucose levels. Their study showed that removing liver kinase B1 (LKB1) in the liver did not hinder AMPK activation in muscle tissue but did eliminate metformin’s impact on serum glucose levels. This suggests that, in mice, metformin primarily reduces blood glucose concentrations by suppressing hepatic gluconeogenesis. Furthermore, the study suggests that LKB1 acts as a tumor suppressor, indicating that increased CREB-dependent or SREBP-1-dependent transcription may contribute to LKB1-related tumorigenesis. These findings highlight the intricate link between the physiological regulation of metabolism and cancer. The mTOR, insulin, and LKB1 pathways collectively form a fundamental eukaryotic network that governs cell growth in response to environmental nutrients. Dysregulation of any of these pathways can contribute to the development of both diabetes and cancer [76].

#### 3.6.4. Bariatric Surgery

In a study involving a cohort of 1,045,348 patients with obesity between 50 and 74 years old, compared with the general population, individuals of the same age with obesity who did not undergo bariatric surgery had an increased risk (34%) of developing CRC [81]. Bariatric-surgery-induced reductions in systemic inflammation and hyperinsulinemia may contribute to the protective effect against CRC development [60].

Surgical obesity treatment might be more effective in preventing cancer development than medical therapy. When comparing medical therapy to surgery for obesity, a prospective study revealed that 15-year surgical weight loss was as high as 27% for gastric bypass patients, compared with minimal weight loss in the medically treated group [7]. Metabolic gastrointestinal operations can produce sustained long-term resolution of diabetes [77]. Surgical patients have a five times higher remission rate for diabetes in patients with obesity compared with medical controls [78].

**Figure 2:**
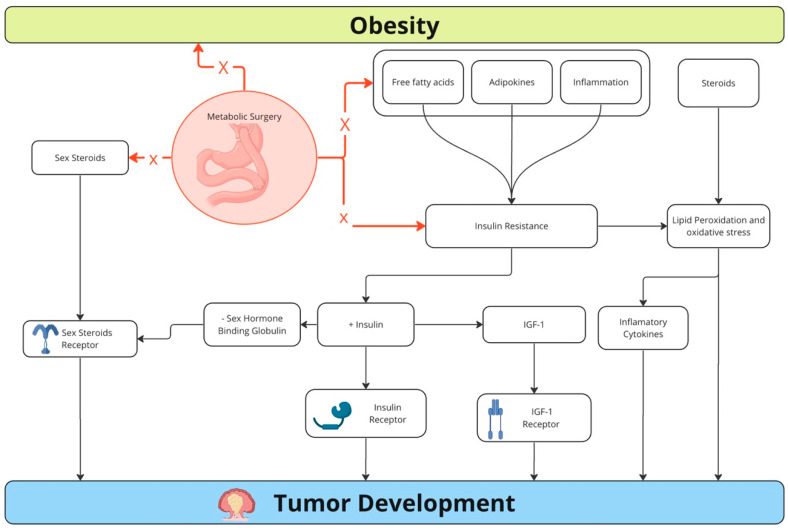
Mechanisms of decreased cancer risk by metabolic surgery. X = acts by blocking or reducing.

Hormonal alterations subsequent to bariatric surgery decrease the risk of CRC, particularly in tumors expressing the estrogen receptor β [79]. Besides, metabolic surgery decreases oxidative stress and systemic inflammatory markers, including IL-6, C-reactive protein (CRP), and plasminogen activator inhibitor-1. Multiple regression analysis reveals that decreased postoperative insulin resistance after surgery is independently associated with decreased IL-6 concentrations. Six months after surgery, there is also a change in the production of interferon-gamma, IL-12, and IL-18, associated with a change in natural killer (NK) cell activity. This may suggest a role for metabolic surgery in contributing to a cell-mediated cytotoxic immune response against tumor cells to achieve surgical anticancer effects [7]. The decrease in leptin production by adipocytes post-surgery also contributes to decreased tumor rates, as leptin is known to stimulate carcinogenesis [80]. Furthermore, metabolic surgery significantly reduces ghrelin levels, a hormone with carcinogenic properties, particularly in neuroendocrine tumors, gastrointestinal tumors, and prostate cancer [7]. Figure 2 summarizes the main mechanisms of metabolic surgery to reduce the risk of cancer development. 

However, various bariatric procedures may have differing impacts on CRC risk. Variations in the effectiveness of weight loss, alterations in the gut microbiome, malabsorption, or changes in circulating gastrointestinal hormones could result in heterogeneous rates of cancer risk reduction.

### 3.7. Treating Patients with Obesity and Colorectal Cancer

For patients with diagnosed CRC, BMI-controlling strategies, such as exercise, diet control, nutritional therapy, and medications, are beneficial for patients with obesity. Managing BMI helps CRC patients control their weight, reducing the incidence of adverse events during treatment [7,56]. Preoperative intentional weight loss in patients with excess weight awaiting colorectal cancer surgery may reduce postoperative morbidity by enhancing physical function and cardiovascular fitness, reducing systemic inflammation, and promoting glucose regulation [56]. Weight loss might help enhance the effectiveness of chemo- and radio-therapy and reduce the risk of surgical procedures.

Various energy-balance components, encompassing physical activity and body composition, have been independently associated with CRC survival and recurrence. Recent research indicates that individuals engaging in moderate physical activity may experience a protective effect, particularly beneficial for patients with CRC and high BMI, potentially improving their prognosis. Consistent and moderate physical activity amplifies basal metabolism and augments tissue oxygenation, enhancing outcomes for individuals fighting CRC [81]. Patients engaging in 18 or more metabolic equivalent task (MET) hours per week demonstrate a remarkable 52% reduction in the risk of death or recurrence. Achieving the recommended 18 MET hours per week is feasible, whether through 90 min of weekly running or a dedicated 6 h of weekly walking [82].

Successful weight loss in patients with obesity and cancer relies on several key factors. Firstly, psychological support plays a crucial role in addressing anxiety and mood disorders common in cancer patients [83]. This support helps patients cope with the emotional challenges of the cancer diagnosis and treatment, improving their ability to adhere to weight loss interventions. Secondly, acquiring knowledge about obesity and postoperative complications is essential. Educating patients about the risks associated with obesity and the benefits of weight loss can motivate them to make necessary lifestyle changes. Lastly, family support is essential. A supportive home environment can encourage patients to adhere to their weight loss plans and make healthier choices [83].

There is currently no specific target for weight loss before initiating cancer treatment for CRC. The optimal approach to weight loss in this context remains uncertain, as there is a lack of clear evidence regarding the best methods and the amount of weight that should be targeted. In this setting, patients must receive support from a specialized nutritional and physical rehabilitation team. Such a team can provide individualized guidance and support, identifying patients’ vulnerabilities and demands and helping patients make informed decisions about their weight loss goals and strategies. This support can help optimize patients’ overall health and fitness before cancer treatment, improving treatment outcomes. As mentioned earlier, malnutrition is associated with a poor prognosis. Therefore, weight loss strategies before cancer treatment should be carefully considered and always supported by a specialized team.

Finally, while current evidence is lacking for bariatric surgery in the pretreatment period of cancer, patients with rectal cancer who are planning for neoadjuvant therapy could potentially benefit from a bariatric procedure. During the neoadjuvant therapy period, the patient is likely to lose weight, which may improve their fitness for surgical resection. Due to the lack of evidence supporting this strategy, this approach should be considered cautiously and limited to specialized centers, following multidisciplinary meetings and shared decision-making with the patient and their family.

### 3.8. Role of Future Studies

High-quality basic research and large-scale multicenter prospective studies are still needed to explore the relationship between CRC and obesity. Determining the optimal weight loss target to achieve the best cancer outcomes with minimal complications is crucial. Additionally, investigating the role of different types of bariatric surgery techniques as a component of colorectal cancer treatment is essential, ensuring that the strategy improves survival rates without leaving patients at risk for malnutrition before cancer resection.

## 4. Conclusions

In conclusion, addressing obesity is paramount for reducing the risk of CRC development and improving treatment outcomes. Obesity is intricately linked to carcinogenesis, making it a crucial target for intervention. A comprehensive approach to managing obesity is essential for reducing the burden of CRC and improving patients’ outcomes.

## Figures and Tables

**Table 1:** Search strategy summary.

Items	Specification
Date of search	Last search was conducted on 29 February 2024.
Databases and other sources searched	The search encompassed databases such as PubMed, Embase, Lilacs/BVS, Cochrane Central, and Google Scholar.
Search terms used	Search terms included “cancer”, “neoplasm”, “tumor”, “oncogenesis”, “oncology”, “obesity”, “obese”, “overweight”, “insulin resistance”, “metabolic syndrome”, “colorectal”, “colonic”, “colon”, “rectal”, and “rectum”.
Timeframe	Articles were considered for inclusion from inception of these databases until February 2024.
Inclusion and exclusion criteria	Only English and Portuguese studies were considered for inclusion. The review encompassed observational and experimental studies, including human studies, in vivo and in vitro studies, and animal models.
Selection process	A non-systematic study selection was independently conducted by two authors (B.C.J.M. and E.T.N.). Any disagreement regarding inclusion was solved by a third experienced author (F.T.).
